# Kinetics of the SARS-CoV-2 Antibody Avidity Response Following Infection and Vaccination

**DOI:** 10.3390/v14071491

**Published:** 2022-07-08

**Authors:** Laura Garcia, Tom Woudenberg, Jason Rosado, Adam H. Dyer, Françoise Donnadieu, Delphine Planas, Timothée Bruel, Olivier Schwartz, Thierry Prazuck, Aurélie Velay, Samira Fafi-Kremer, Isabella Batten, Conor Reddy, Emma Connolly, Matt McElheron, Sean P. Kennelly, Nollaig M. Bourke, Michael T. White, Stéphane Pelleau

**Affiliations:** 1 Infectious Diseases Epidemiology and Analytics Unit, Department of Global Health, Institut Pasteur, Université Paris Cité, 75015 Paris, France; laura.garcia@pasteur.fr (L.G.); tom.woudenberg@pasteur.fr (T.W.); javier.rosado@pasteur.fr (J.R.); francoise.donnadieu@pasteur.fr (F.D.); 2 Tallaght University Hospital, Tallaght, D24 NR0A Dublin, Ireland; dyera@tcd.ie (A.H.D.); sean.kennelly@tuh.ie (S.P.K.); 3 Department of Medical Gerontology, School of Medicine, Trinity College Dublin, D02 PN40 Dublin, Ireland; batteni@tcd.ie (I.B.); reddyco@tcd.ie (C.R.); econnol7@tcd.ie (E.C.); mcelherm@tcd.ie (M.M.); nbourke@tcd.ie (N.M.B.); 4 Virus & Immunity Unit, Department of Virology, Institut Pasteur, Université Paris Cité, 75015 Paris, France; delphine.planas@pasteur.fr (D.P.); timothee.bruel@pasteur.fr (T.B.); olivier.schwartz@pasteur.fr (O.S.); 5 CHR d’Orléans, Service de Maladies Infectieuses, 45100 Orléans, France; thierry.prazuck@chr-orleans.fr; 6 CHU de Strasbourg, Laboratoire de Virologie, CEDEX, 67091 Strasbourg, France; aurelie.velay@chru-strasbourg.fr (A.V.); samira.fafi-kremer@unistra.fr (S.F.-K.); 7 Unité Mixte de Recherche Scientifique Immuno-Rhumathologie Moléculaire (IRM UMR-S) 1109, Strasbourg University, Institut National de la Santé et de la Recherche Médicale (INSERM), CEDEX, 67084 Strasbourg, France

**Keywords:** SARS-CoV-2, serology, multiplex, antibody, avidity, kinetics, time since infection

## Abstract

Serological assays capable of measuring antibody responses induced by previous infection with severe acute respiratory syndrome coronavirus 2 (SARS-CoV-2) have been critical tools in the response to the COVID-19 pandemic. In this study, we use bead-based multiplex assays to measure IgG and IgA antibodies and IgG avidity to five SARS-CoV-2 antigens (Spike (S), receptor-binding domain (RBD), Nucleocapsid (N), S subunit 2, and Membrane-Envelope fusion (ME)). These assays were performed in several cohorts of healthcare workers and nursing home residents, who were followed for up to eleven months after SARS-CoV-2 infection or up to six months after vaccination. Our results show distinct kinetic patterns of antibody quantity (IgG and IgA) and avidity. While IgG and IgA antibody levels waned over time, with IgA antibody levels waning more rapidly, avidity increased with time after infection or vaccination. These contrasting kinetic patterns allow for the estimation of time since previous SARS-CoV-2 infection. Including avidity measurements in addition to antibody levels in a classification algorithm for estimating time since infection led to a substantial improvement in accuracy, from 62% to 78%. The inclusion of antibody avidity in panels of serological assays can yield valuable information for improving serosurveillance during SARS-CoV-2 epidemics.

## 1. Introduction

Severe acute respiratory syndrome coronavirus 2, or SARS-CoV-2, emerged as a zoonotic virus and was identified as the causative agent of COVID-19 in December 2019. SARS-CoV-2 is a Betacoronavirus belonging to the Sarbecovirus subgenus, like SARS-CoV. Coronaviruses have a positive-sense RNA genome of 26–32 kilobases. This genome encodes four structural proteins: Spike (S), Nucleocapsid (N), Envelope (E) and Membrane (M). The most important for protective immunity is the glycoprotein Spike, which forms a trimeric structure on the virus surface and comprises two subunits. Spike subunit 1 (S1) contains the receptor-binding domain (RBD) responsible for binding to the angiotensin-converting enzyme 2 (ACE2) receptor on the host cell, while Spike subunit 2 (S2) permits the fusion of the viral and cellular membranes. Nucleocapsid plays an important role in transcription enhancement and viral assembly.

The kinetics of the SARS-CoV-2 antibody response following infection or vaccination have been analyzed in detail, with numerous studies demonstrating that specific immunoglobulin antibodies (IgG, IgA and IgM) to SARS-CoV-2 antigens develop between 6–15 days following symptom onset or vaccination. Following an initial period of boosting, antibody levels wane rapidly within the first 3–6 months, followed by a transition to a more slowly waning phase [1,2,3]. The different phases in the kinetics of the antibody response can be explained by a balance between populations of antibody-secreting plasma B cells with a short half-life (predominantly in the spleen) and a long half-life (located in the bone marrow). Over time, memory B cells increasingly differentiate into long-lived plasma cells present in the bone marrow, leading to a more mature antibody response [4]. Long-term follow-up of individuals infected with SARS-CoV-1 has shown that antibodies remain detectable six years after infection but continue to decrease [5].

Affinity maturation is the biological mechanism by which activated B cells undergo rounds of somatic hypermutations in immunoglobin genes, followed by an iterative clonal selection in germinal centers, resulting in the production of antibodies with greater affinities to the antigens over time [6,7]. Structural changes consist of slight amino acid mutations in the variable domains of antibodies, which improves the conformational fit of antibodies into their binding sites, therefore increasing the stability of the immune complexes. Antibody avidity, or functional affinity, measures the total strength of all of the non-covalent interactions between an antibody and its target antigen and can be extended to the total antigen-binding force of antibodies specific to a given antigen in sera. Avidity depends on three parameters: firstly, the binding affinity of the complex of antibodies and the antigen via a non-covalent interaction; secondly, the valency of the antibody; and thirdly, the structural arrangement of the antibody and antigen in the complex. While antibodies with low avidity are produced during the primary response, the progressive increase in the avidity of antibodies over time hence constitutes a useful marker of the maturation of the immune response and could help in providing estimates of time since infection.

In addition to providing insight into the immunology of SARS-CoV-2 infection, the measurement of antibody responses can provide valuable epidemiological information through the implementation of seroprevalence surveys [8]. In the case of SARS-CoV-2, the majority of seroprevalence studies involve the measurement of anti-N or anti-S IgG responses using immunoassays such as enzyme-linked immunosorbent assays (ELISA). In 2020, before the widespread roll-out of vaccines to prevent COVID-19, serological tests based on anti-N or anti-S IgG were demonstrated to have high sensitivity and high specificity for identifying individuals previously infected with SARS-CoV-2 [9]. However, the waning of antibodies was associated with substantial reductions in diagnostic sensitivity over time. The roll-out of COVID-19 vaccines has altered the role of seroprevalence surveys, as assays based on Spike proteins now measure a combination of naturally acquired and vaccine-induced immunity. Measurement of anti-N IgG can be used to distinguish naturally acquired from vaccine-induced immunity; however, this is complicated by the short duration of anti-N IgG antibodies [3], resulting in varying durations of seropositivity following infection [10].

In contrast to monoplex assays such as ELISA, multiplex serological assays can simultaneously measure antibodies to multiple antigens, allowing for more epidemiological information to be obtained from a single test. It has been demonstrated that multiplex assays can have higher accuracy than monoplex assays [11,12]; can distinguish vaccinated from naturally infected individuals [13]; can provide estimates of the time since previous infection [3]; and can simultaneously measure immunity to seasonal coronaviruses [14].

The majority of public health applications of serological assays involve the measurement of IgG or IgM antibodies. However, due to the distinct kinetic profiles of antibody responses, there are some notable examples where measuring avidity provides important additional clinical or epidemiological information. Avidity assays can be used to distinguish recent HIV infections from older infections [15], to diagnose cytomegalovirus or rubella viruses during pregnancy [16,17] and to identify cases of measles infection following vaccine failure [18,19]. In this multicentric study, we performed a detailed analysis of the kinetics of the antibody avidity response following infection or vaccination and demonstrated how antibody avidity can be used to improve the estimation of the time since previous SARS-CoV-2 infection.

## 2. Materials and Methods

### 2.1. Samples

The samples used in this study are summarized in Table 1. A panel of 522 positive serum samples from 174 healthcare workers with a proven history of SARS-CoV-2 infection in Strasbourg hospitals were collected, with up to three samples per individual collected over a period of nine months following symptom onset [20]. A second panel of samples was collected from healthcare workers in Institut Mutualiste Montsouris (IMM), a Parisian hospital. Of 784 healthcare workers sampled in April 2020, 32 were identified as having previous SARS-CoV-2 infection. For 29/32 of these healthcare workers, an additional sample was collected in February 2021. The longest duration of follow-up after infection in this study was 11 months. The 752 healthcare workers from IMM who were not infected before April 2020 formed the negative group for our study. A pool of serum from 27 PCR-positive healthcare workers from IMM was used for assay calibration.

In a study of vaccine-induced immune responses, 86 residents of nursing homes in Dublin, Ireland, were followed before and after receiving two doses of the Pfizer BNT162b2 vaccine [21]. Samples were collected at baseline and 5 weeks and 6 months after the second dose. Thirty-nine individuals had prior SARS-CoV-2 infection, and forty-seven were SARS-CoV-2 naïve. Another prospective, longitudinal cohort was established in the French city of Orléans to study immunity to SARS-CoV-2 following infection or vaccination. Sixteen individuals who received the Pfizer two-dose vaccine regimen were followed for up to 168 days.

### 2.2. Serological Assay

A previously described 9-plex bead-based assay was used for simultaneous detection of antibodies to 5 SARS-CoV-2 antigens and 4 seasonal coronaviruses (spike proteins of NL63, 229E, HKU1 and OC43) in 1 μL serum or plasma samples [3]. SARS-CoV-2 antigens of the ancestral lineage were from Spike (whole trimeric Spike, its RBD and S2), Nucleocapsid protein and Membrane-Envelope fusion protein (ME). ME and S2 antigens were purchased from Native Antigen (Oxford, United Kingdom), and all other antigens were produced as recombinant proteins at Institut Pasteur. The mass of proteins coupled to beads was optimized to generate a log-linear standard curve with a pool of 27 positive sera prepared from patients with reverse-transcription quantitative PCR–confirmed SARS-CoV-2. We measured the levels of immunoglobulin G (IgG) and immunoglobulin A (IgA) of each sample in two separate assays. Plates were read using a Luminex MAGPIX^®^ system, and the median fluorescence intensity (MFI) was used for analysis. A 5-parameter logistic curve was used to convert MFI to relative antibody units relative to the standard curve generated on the same plate to account for inter-assay variation.

### 2.3. Multiplex Avidity Assay

To study avidity, we used a single concentration of a chaotropic agent to destabilize antibodies bound to antigens and measured the proportion of antibodies that remained bound after treatment in order to obtain a relative measure of the total binding strength of antibodies. The protocol was optimized by comparing the effects and dynamic range of three chaotropic agents (urea, guanidine hydrochloride and ammonium thiocyanate) on avidity measurements and testing their side effects on target antigens coupled to beads, incubation times and concentrations. Following assay optimization, a routine avidity assay was performed by treating serum samples at a single concentration of urea at 6M. The protocol for the avidity assay was similar to that of the serological assay with the inclusion of an additional step. After the incubation of beads with serum samples, the bead–Ab complexes were washed with assay buffer PBT (PBS-Tween20 0.05%-BSA 1%) and then incubated for 5 min with 100 μL of urea 6M diluted in water or water alone as a control. After 3 washing steps, 100 μL of anti-IgG secondary antibody conjugated to R-phycoerythrin (Jackson Immunoresearch) diluted at 1/100 was added for 15 min. After 3 final washes to remove unbound secondary antibodies, plates were read using a Luminex^®^ MAGPIX^®^ system, and the median fluorescence intensity (MFI) was used for analysis. Avidity was only assayed for IgG.

### 2.4. Statistical Analysis

All statistical analyses were performed with R version 4.0.5. Median fluorescence intensities (MFIs) were converted to relative antibody units (RAU) with a 5-parameter logistic curve relative to the standard curve generated on the same plate. The avidity index (AI) was calculated as the MFI of the sample treated with the chaotropic agent divided by the MFI without the chaotropic agent times 100. Linear regression was used to compare the difference between the means of the different groups of time since infection with SARS-CoV-2.

To assess whether avidity is of additional value to antibody measurements in the estimation of time since previous SARS-CoV-2 infection, we predicted time since infection with antibody measurements only and with antibody measurements and avidity estimates. We developed two random forest regression models. For each random forest, the number of trees was set at 1000. For each tree, two-thirds of the observations were used. Predictions were derived from the average of samples’ estimates in the remaining one-third of the samples, the out-of-bag samples. Regressions were built in a step-wise manner. First, the antigen in the regression was selected based on the importance of that antigen, measured by the mean decrease in accuracy on the out-of-bag samples. Subsequently, all other variables were added one by one to the most important antigen in the regression. The antigen associated with the lowest residual sum of squares was kept in the model. This process was repeated until no further decrease in the lowest residual sum of squares was observed. The randomForest package was used to develop and evaluate the random Forest regression models [22].

## 3. Results

### 3.1. Kinetics of SARS-CoV-2 Antibody Levels

**Figure 1:**
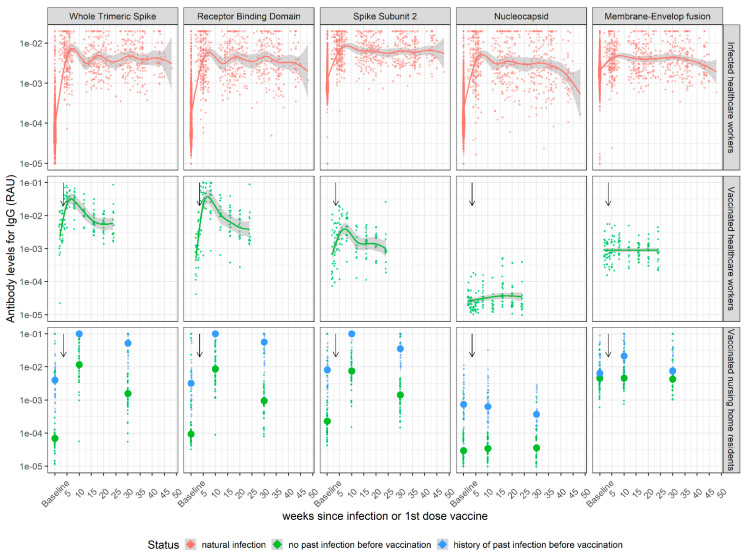
IgG antibody kinetics following SARS-CoV-2 infection or vaccination with BNT162b2. IgG antibodies to five SARS-CoV-2 antigens were measured in serum samples using a bead-based multiplex Luminex assay. (First row) Healthcare workers from hospitals in Strasbourg and Paris were followed longitudinally after PCR-confirmed SARS-CoV-2 infection. (Middle row) Healthcare workers from a hospital in Orléans were followed longitudinally after receiving two doses of Pfizer BNT162b2 vaccine. (Bottom row) Residents of a nursing home in Dublin were followed after receiving two doses of Pfizer BNT162b2 vaccine. Individuals with “history of past infection” correspond to individuals with recorded SARS-CoV-2 infection before vaccination and are represented with blue dots. Individuals with no history of past infection are in green. Time is denoted as weeks post-vaccination. Thicker dots represent the median of each group. Black arrows indicate the date of the second vaccine injection.

We evaluated SARS-CoV-2-specific antibody responses over time in healthcare workers and care home residents following PCR-confirmed SARS-CoV-2 infection or administration of Pfizer’s BNT162b2 vaccine (Table 1, 308 individuals and 938 samples). IgG antibody kinetics showed a consistent profile, with antibody levels increasing sharply following infection or vaccination, followed by an initial phase of rapid waning over the first 3–6 months and then by a transition to a phase of slower waning (Figure 1). IgA directed against whole Spike and RBD antigens followed a qualitatively similar pattern to IgG kinetics, with the exception that IgA antibodies waned more rapidly than IgG antibodies (Figure S1). Of note, for individuals who were vaccinated and had no history of past infection, no significant antibody signals to Nucleocapsid or Membrane-Envelope antigens were detected, as expected. Compared to nursing home residents with no history of natural infection, residents who were infected before vaccination showed a qualitatively similar kinetic pattern but consistently higher IgG responses to whole Spike, RBD and S2 antigens at all time points. 

### 3.2. Kinetics of SARS-CoV-2 IgG Antibody Avidity

**Figure 2:**
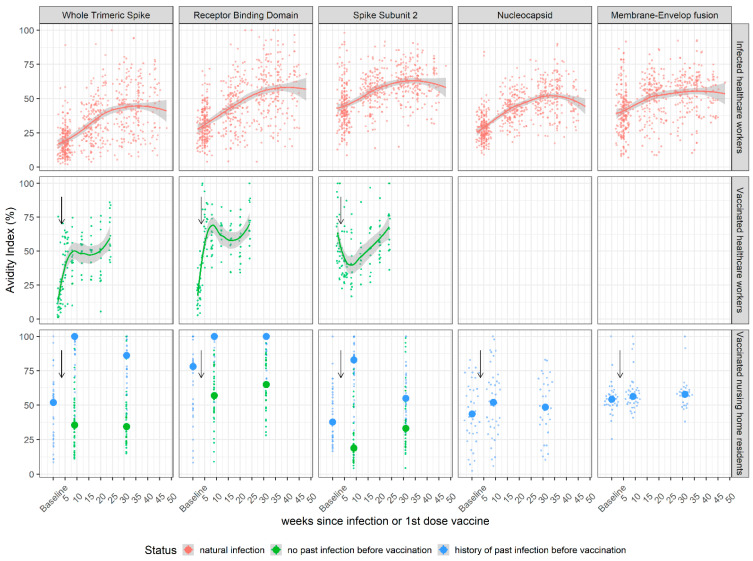
Kinetics of IgG avidity following SARS-CoV-2 infection or vaccination with BNT162b2. IgG avidity to five SARS-CoV-2 antigens was measured in serum samples using a bead-based multiplex Luminex assay. (First row) Healthcare workers from hospitals in Strasbourg and Paris were followed longitudinally following PCR-confirmed SARS-CoV-2 infection. (Middle row) Healthcare workers from a hospital in Orléans were followed longitudinally after receiving two doses of Pfizer BNT162b2 vaccine. (Bottom row) Residents of a nursing home in Dublin were followed after receiving two doses of Pfizer BNT162b2 vaccine. Individuals with “history of past infection” correspond to individuals with recorded SARS-CoV-2 infection before vaccination and are represented with blue dots. Individuals with no history of past infection are in green. Time is denoted as weeks post-vaccination. Thicker dots represent the median of each group. Black arrows indicate the date of the second vaccine injection. The avidity indexes of anti-Nucleocapsid and anti-Membrane-Envelope IgG are not shown for unvaccinated individuals, as well as data points for anti-spike (whole spike, RBD and S2) IgG of nursing home residents with no prior history of infection before vaccination.

Analysis of the kinetics of the SARS-CoV-2 IgG antibody avidity response revealed a general pattern of increasing avidity over the monitored timeframe for both healthcare workers who were vaccinated or naturally infected (Figure 2, top and middle rows). However, this rise in avidity index showed a slightly different profile in vaccinated or naturally infected individuals for spike-related antigens. The median avidity index (AI) of anti-RBD IgG in vaccinated individuals reached a peak at 60% only 10 weeks after the first vaccine dose, while it took 30 weeks to reach an equivalent median AI in naturally infected individuals. Among elderly individuals, a significantly different kinetic profile was observed according to the history of natural infection preceding vaccination. Individuals who were infected and vaccinated (Figure 2, bottom panel, blue dots) showed a very homogeneous response with a strong increase in AI 10 weeks after vaccination that reached a peak median AI at 100% for whole spike and RBD antigens. In contrast, nursing home residents who were vaccinated with no prior infections (Figure 2, bottom panel, green dots) showed a median AI similar to or slightly lower than vaccinated HCWs (blue dots). Finally, the median AI of anti-N and anti-ME IgG among vaccinated individuals with prior infection (Figure 2, bottom panel, blue dots) remained stable throughout the study period and was therefore not influenced by vaccination.

### 3.3. Estimation of Time since Infection

**Figure 3:**
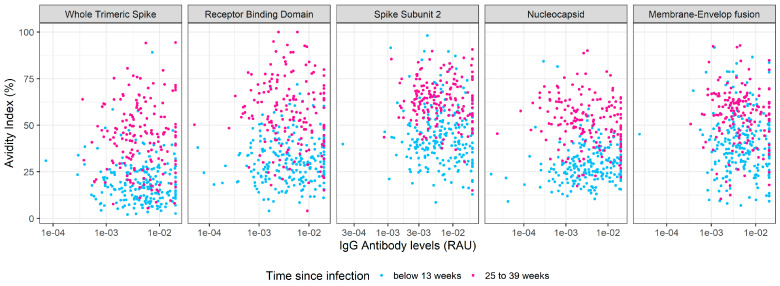
Serological markers of time since infection. Anti-SARS-CoV-2 IgG levels and avidity were measured in samples from individuals with recent (within the previous 3 months, red) and older (6–9 months ago, blue) naturally acquired SARS-CoV-2 infection.

The clear temporal trends in antibody avidity demonstrate that there is a statistical signal for estimating time since previous infection. Figure 3 shows a comparison of anti-SARS-CoV-2 IgG levels and avidity for individuals with recent (less than 3 months) and older (6–9 months ago) naturally acquired SARS-CoV-2 infection. This representation shows that the two populations can be visually distinguished.

**Figure 4:**
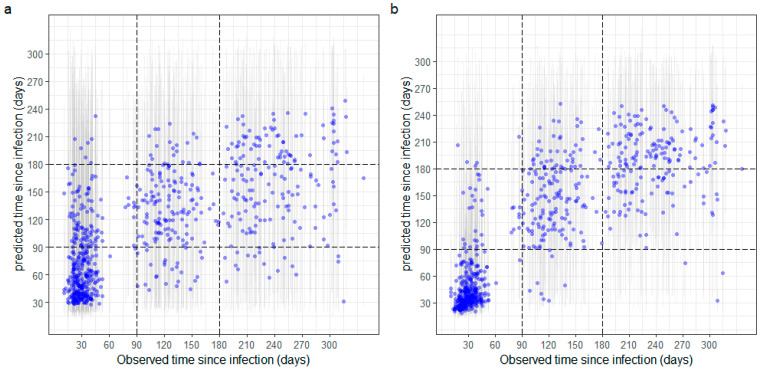
Predictions of time since infection without (**a**) and with (**b**) avidity measurements. Predictions are derived from random forest regression models, with point estimates in blue and 95% uncertainty intervals as vertical bars. (**a**) Predictions without avidity measurements from a random forest model with 6 biomarkers: NP IgA, S IgA, NP IgG, S2 IgA, RBD IgA and S2 IgG. (**b**) Predictions with avidity measurements from a random forest model with 6 biomarkers: NP avidity, NP IgA, NP IgG, S avidity, RBD avidity and S2 avidity.

Estimation of time since infection was conducted with two random forest regression models. In the first model, we considered IgG and IgA antibodies. In the second model, we also included avidity measurements. The regression model without avidity estimates showed that RAU to NP IgA provided the highest accuracy, followed by NP IgG and RBD IgA (Figure S2a). The final model predicting time since infection without avidity included the following biomarkers: NP IgA, Spike IgA, NP IgG, S2 IgA, RBD IgA and S2 IgG. With these antigens, the regression model yielded a residual sum of squares of 4788 (Figure S2b). Categorizing each prediction into the categories of 3 months and less, 4 to 6 months, and infections longer than 6 months ago could classify 62.5% of the samples correctly (Figure 4a). Combining antibody levels and avidity led to improved accuracy of time since infection estimates, with the most informative biomarkers being NP avidity followed by Spike avidity. Antigens that reduced the residual sum of squares to 2608 were NP avidity, NP IgA, NP IgG, Spike avidity, RBD avidity and S2 IgA (Figure S2c). The predictions of this regression model are shown in Figure 4b. When we categorized the predictions into the three categories, 78% of the classifications were correct: 90% of samples taken within 3 months after symptom onset were classified correctly, 69% of samples 4 to 6 months were classified correctly, and 63% of samples taken 6 to 9 months after symptom onset were classified correctly.

## 4. Discussion

COVID-19 remains a critical threat to public health, and a deeper understanding of how antibodies are produced during infection is crucial. As can be seen from our results and others [23,24,25], antibody kinetics differ according to isotype and target antigen. Indeed, IgA wanes more rapidly than IgG antibodies for the studied SARS-CoV-2 antigens. This has potential implications for the duration of protective immunity against SARS-CoV-2 infection. The effect of the antibody level on immunity to SARS-CoV-2 has been well studied, with anti-Spike IgG levels and neutralizing antibody titers shown to be correlated and associated with protection from infection in vaccine studies [26,27,28]. The effect of the quality of the antibody response to SARS-CoV-2 has received comparatively less attention. As avidity measures the overall binding strength between antibodies and antigens, it has been suggested that avidity may be associated with protection against infection and has the potential to complement antibody titer data in the search for biological correlates of clinical protection [29].

The rationale is that for an equivalent number of antibodies, avidity can make a difference in terms of the quality of the response. Similarly, declining levels of antibodies over time could be partly compensated by an increase in avidity, thus maintaining an equivalent or even superior protective efficacy compared to serum with high antibody titers with poor avidity. This phenomenon has been observed in studies of vaccines against other pathogens, such as *Plasmodium falciparum* malaria [30] or *Streptococcus pneumonia* [31], where both antibody levels and avidity were shown to be significantly associated with vaccine efficacy.

In accordance with other studies [32], we found a progressive increase in IgG avidities to SARS-CoV-2 antigens in convalescent and vaccinated individuals after exposure. Although both groups were able to reach similar levels, we observed a significant difference in the rate at which this response took place. The median avidity index of vaccinated individuals exceeded 50% three times faster than the avidity index developed by naturally infected individuals (10 vs. 30 weeks). We could observe the same rapid boost in antibody avidity 10 weeks after vaccination in individuals who were previously infected, up to the maximal value of 100%. Other studies have reported a similar net increase in antibody avidity in previously exposed individuals receiving a vaccine booster dose, which was associated with a more efficient binding inhibition of spike to ACE2 in an in vitro competition assay [33]. It would be interesting to investigate whether this steeper increase in avidity associated with vaccination is caused by an acceleration of affinity maturation in germinal centers or simply reflects an ongoing trajectory towards a more complete process of affinity maturation. While our data seem to indicate the stabilization of anti-RBD IgG avidity at the end of follow-up in healthcare workers and care home residents, actually, it would be helpful to extend this follow-up to see how it evolves and correlates with neutralization efficiency.

In addition to being a critical tool for understanding the determinants of clinical protection, serological data can also provide important epidemiological information. Antibody avidity assays can be useful for the diagnosis of recent infections, as previously demonstrated for other pathogens such as measles, CMV or HIV [15,16,18]. The main objective of our study was to see whether avidity data could improve our previous model relying on IgG and IgA levels to spike and nucleocapsid antigens to produce estimates of time since infection based on its distinct kinetic pattern. By using machine learning algorithms to combine the data types, we conclude that antibody avidity is an even more accurate biomarker for identifying recent infection and leads to a substantial improvement to 78% accuracy.

One concern in the current situation is the continuous emergence and spread of SARS-CoV-2 variants characterized by antigenic changes in the Spike protein. These mutations are associated with reduced susceptibility to infection- or vaccine-induced immunity. Impaired binding efficiency of IgG to mutant RBD epitopes and enhanced stability of the ACE2-RBD complex [34] induced by key changes, such as mutation of residue 484, are two complementary mechanisms that would be very interesting to quantify at the epidemiological level with avidity measures in order to better know the determinants of transmission dynamics and the correlates of protection. In particular, it would be useful to study how these differential avidity responses to new variants can impact our prediction model and help to reconstruct successive waves of infection with variants of concerns.

Altogether, we provide further evidence that the integration of the IgG avidity parameter in the serological toolkit combined with epidemiological models can play a useful role in the response to the COVID-19 pandemic.

## Figures and Tables

**Table 1:** Panels of samples included in the study.

	Natural Infection			Vaccination		
	Strasbourg HCWs	Paris HCWs; Infected	Paris HCWs; Uninfected	Orléans HCWs	Dublin CHR; No Past Infection	Dublin CHR; Past Infection
Participants	174	32	752	16	47	39
Samples	522	64	752	120	126	106
Female	139	19	543	5	33	23
Male	35	13	209	11	14	16
Age	43 (25–73)	37 (24–63)	41 (19–72)	59 (35–74)	83 (53–98)	83 (55–100)
Maximum days post-symptom onset	219 (161–284)	304 (285–336)	NA	NA	NA	265 (224–298) *
Days post-vaccination	NA	NA	NA	154 (151–168)	206 (201–210)	206 (201–210)

Abbreviations: HCW, healthcare worker. CHR, care home resident. * Before first sampling.

## Data Availability

All data and code used for reproducing the results are available on request.

## Supplementary Materials

The following supporting information can be downloaded at: https://www.mdpi.com/article/10.3390/v14071491/s1. Figure S1: IgA antibody kinetics following SARS-CoV-2 infection or vaccination with BNT162b2; Figure S2: Model development of random forest regressions predicting time since SARS-CoV-2 infection without and with avidity estimates.
