# Supplementary material for: Kinetics of the SARS-CoV-2 Antibody Avidity Response Following Infection and Vaccination
Source: Viruses. 2022 Jul 8;14(7):1491. doi: 10.3390/v14071491 (PMC9321390; doi:10.3390/v14071491)
Supplement: Supplementary file 1 [file viruses-14-01491-s001.zip › viruses-1726019-supplementary.pdf]

Supplementary Materials

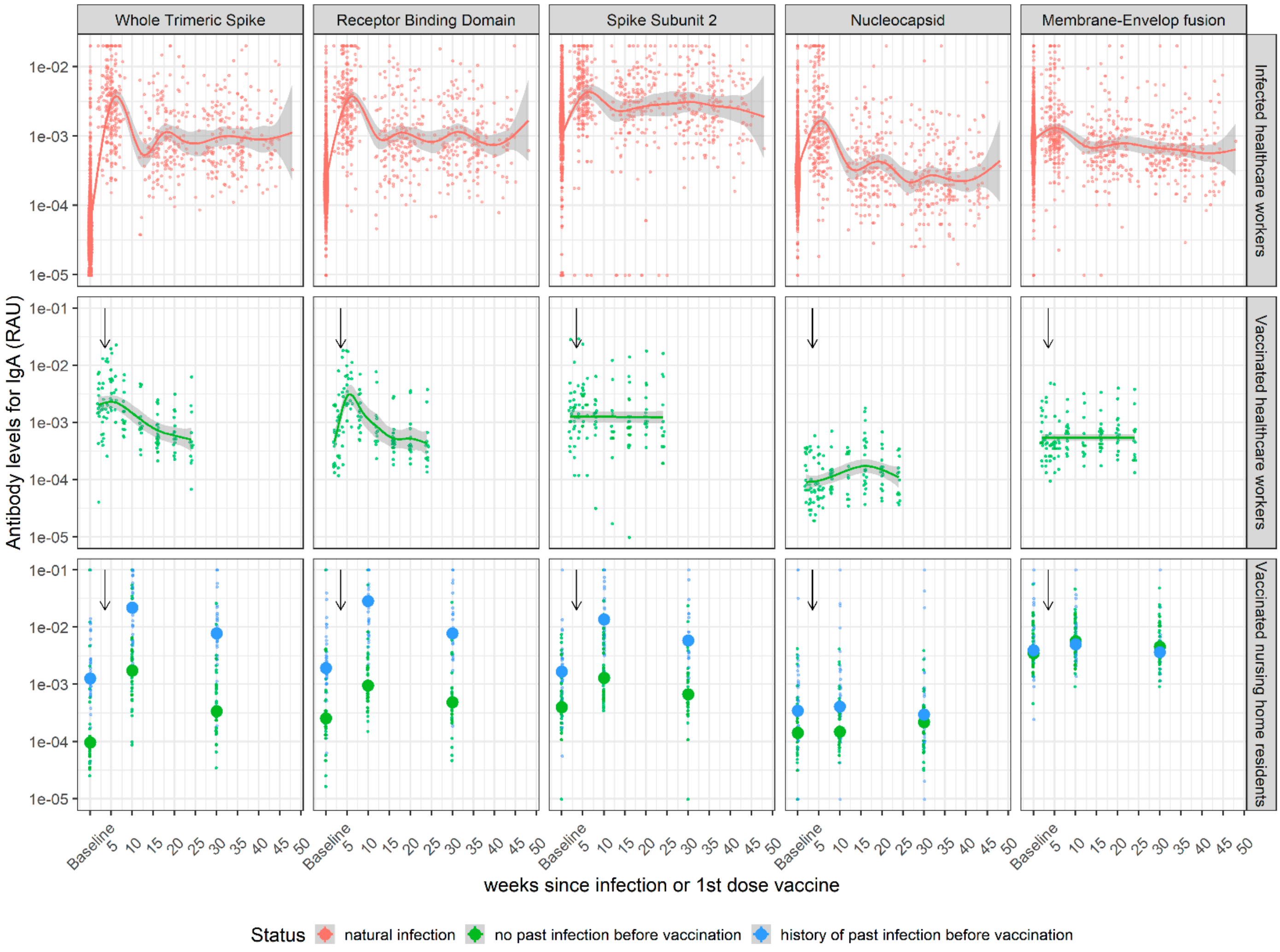

Figure S1: IgA antibody kinetics following SARS-CoV-2 infection or vaccination with BNT162b2. IgA antibodies to five SARS-CoV-2 antigens were measured in serum samples using a bead-based multiplex Luminex assay. (First row) Healthcare workers from hospitals in Strasbourg and Paris were followed longitudinally after PCR–confirmed SARS-CoV-2 infection. (Middle row) Healthcare workers from a hospital in Orléans were followed longitudinally after receiving two doses of Pfizer BNT162b2 vaccine. (Bottom row) Residents of a nursing home in Dublin were followed after receiving two doses of Pfizer BNT162b2 vaccine. Individuals with "history of past infection" correspond to individuals with recorded SARS-CoV-2 infection before vaccination and are represented with blue dots. Individuals with no history of past infection are in green. Time is denoted as weeks post vaccination. Thicker dots represent the median of each group. Black arrow indicates the date of the second vaccine injection.

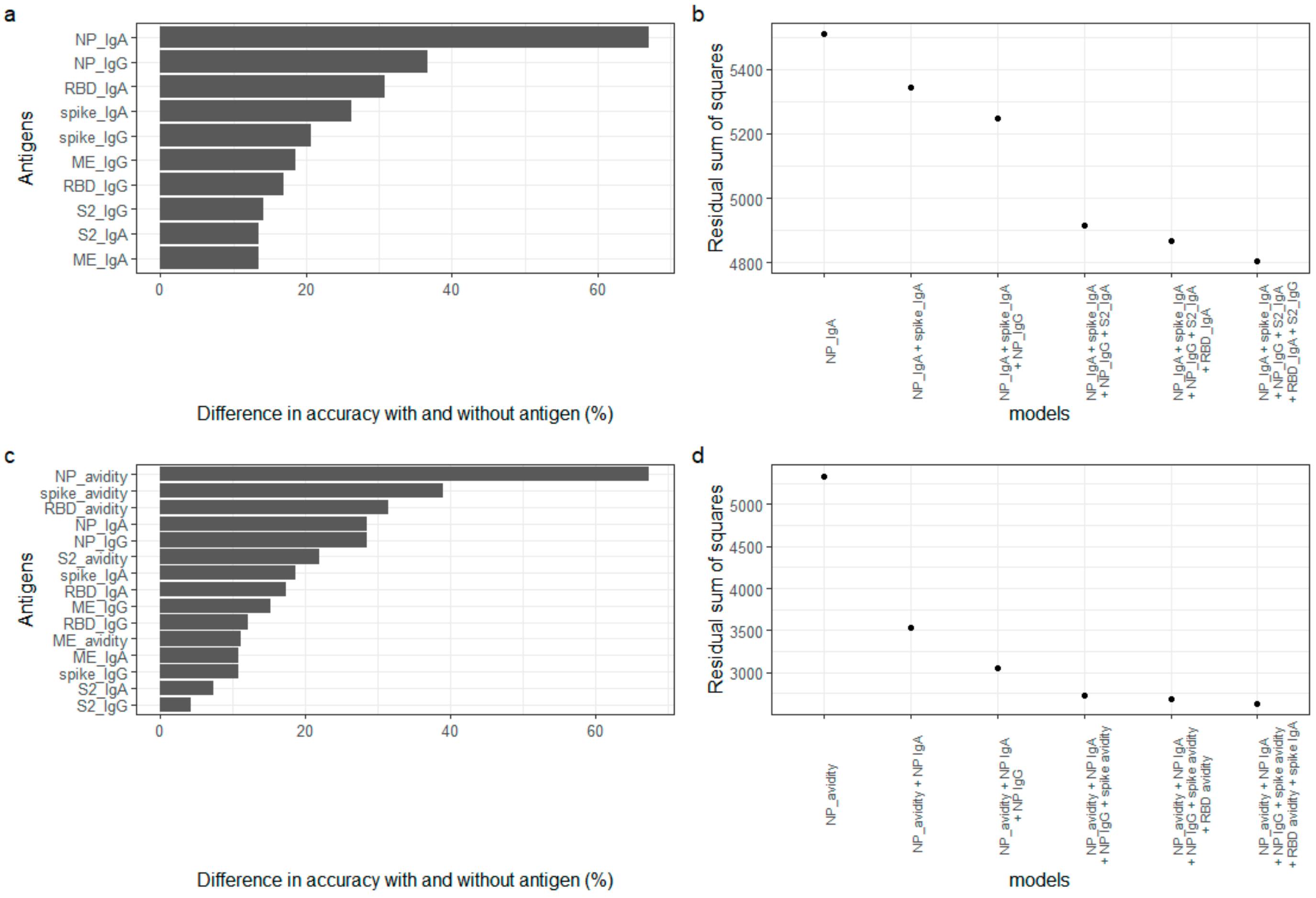

Figure S2: Model development of random Forest regressions predicting time since SARS-CoV-2 infection without (a,b) and with avidity estimates (c,d). **(a)** The variable importance plot provides a ranking of the most important variables in predicting time since SARS-CoV-2 infection. Without avidity estimates, antibodies to NP IgA are most important. **(b)** The stepwise approach of developing a model for antigens to IgA and IgG is visualized by showing the residual sum of squares for each model. **(c).** The most important variable in predicting time since infection was NP avidity followed by Spike avidity and RBD avidity. **(d)** For each model, the corresponding residual sum of squares is shown. Comparison of the residual sum of squares in (b) and (c) shows the better performance of models including avidity variables for estimating time since infection.
